# Endothelial activation and stress index as a prognostic marker in metastatic renal cell carcinoma

**DOI:** 10.3389/fmed.2026.1966150

**Published:** 2026-10-29

**Authors:** Didar Senocak, Eris Ozkan, Mina Karakullukcu, Gizem Ayasgil Ulas, Oguz Bilgi, M. Alpaslan Ozgun

**Affiliations:** Department of Medical Oncology, Sultan II. Abdulhamid Han Training and Research Hospital, University of Health Sciences, Istanbul, Türkiye

**Keywords:** endothelial activation and stress index (EASIX), metastatic renal cell carcinoma, overall survival, prognostic biomarker, systemic inflammation

## Abstract

**Background/objectives:**

The Endothelial Activation and Stress Index (EASIX) is a simple biomarker calculated using LDH, serum creatinine, and platelet count and has shown prognostic significance in various malignancies. However, data on its prognostic role in metastatic renal cell carcinoma (mRCC) are limited. This study investigated the prognostic significance of EASIX in mRCC and compared it with conventional systemic inflammatory indices.

**Methods:**

This retrospective study included 143 patients with mRCC between January 2016 and December 2025. EASIX was calculated using baseline lactate dehydrogenase (LDH), serum creatinine, and platelet count. Exploratory ROC analyses based on observed mortality status were performed to evaluate the discriminatory ability of EASIX, neutrophil-to-lymphocyte ratio (NLR), monocyte-to-lymphocyte ratio (MLR), platelet-to-lymphocyte ratio (PLR), and systemic immune-inflammation index (SII), and to determine a cohort-derived EASIX cut-off value. Overall survival (OS) was assessed using the Kaplan–Meier method and Cox regression analyses were performed to identify factors associated with OS. Additional sensitivity analyses adjusting for ECOG performance status and clinical factors were performed in patients with available ECOG data.

**Results:**

Exploratory ROC analysis of observed mortality identified an EASIX cut-off of 1.293 (AUC: 0.855; 95% CI: 0.796–0.915; *p* < 0.001), with 82.4% sensitivity and 69.6% specificity. ROC analyses of NLR, MLR, PLR, and SII were not statistically significant. Median OS was 22.0 months (95% CI: 14.8–29.2) in the high EASIX group (>1.293) and was not reached in the low EASIX group (≤1.293; log-rank *p* < 0.001). High EASIX remained associated with increased mortality after multivariable adjustment (HR: 4.031; 95% CI: 2.146–7.572; *p* < 0.001) and in ECOG-adjusted sensitivity analysis (HR: 4.149; 95% CI: 1.989–8.653; *p* < 0.001). Liver metastasis (HR: 2.054; 95% CI: 1.152–3.663; *p* = 0.015) and lung metastasis (HR: 1.618; 95% CI: 1.015–2.580; *p* = 0.043) were also associated with worse OS.

**Conclusions:**

High EASIX was associated with worse overall survival in patients with mRCC and demonstrated significant discriminatory ability for observed mortality in exploratory ROC analysis. EASIX may serve as a simple and readily available biomarker for prognostic assessment in mRCC, although the cohort-derived cut-off value requires validation in independent patient populations.

## 1 Introduction

Renal cell carcinoma (RCC) accounts for 2%–3% of adult malignancies and is the most common type of kidney cancer. The incidence of RCC has been increasing in recent years, which is thought to be due to increased incidental detection of renal masses during abdominal imaging performed for nonspecific musculoskeletal or gastrointestinal complaints (1). RCC is closely associated with age and sex, being 1.5 times more common in men than in women. Its incidence increases from the age of 40, and it is most frequently observed between the ages of 60 and 70 (2). While clear cell RCC is the most common histological subtype, papillary, chromophobe RCC, as well as other rare subtypes occurring in less than 1% of cases also exist. Although curative treatment is possible with surgery in localized disease, approximately 15%–17% of patients have distant metastases at the time of diagnosis and require systemic treatment (3). Although survival has significantly improved with targeted therapies and immune checkpoint inhibitors in metastatic disease, the clinical course is quite heterogeneous among patients.

Currently, clinicopathological features such as tumor stage, histological subtype, International Society of Urological Pathology (ISUP)/Fuhrman nuclear grade, performance status, and surgical margins are used as key determinants of prognosis in RCC. However, significant differences in survival outcomes can be observed even in patients with similar stages and histopathological features (4). This situation has increased the need for easily applicable and reliable prognostic markers that can reflect not only tumor biology but also the patient's systemic inflammatory response, endothelial damage, and overall physiological status. One such marker is the Endothelial Activation and Stress Index (EASIX), a simple index calculated from serum lactate dehydrogenase (LDH), serum creatinine, and platelet count that reflects endothelial activation and damage. Each parameter in the EASIX score reflects different aspects of tumor biology. LDH is an indicator of increased anaerobic glycolysis and metabolic reprogramming in tumor cells, and high serum levels are associated with increased tumor burden, rapid cell proliferation, and poor clinical outcomes. Therefore, LDH is one of the key biomarkers contributing to the prognostic power of the EASIX score (5). Serum creatinine levels are considered an indirect indicator of renal function, as well as systemic organ perfusion, microvascular damage, and endothelial dysfunction. Microcirculatory disturbances caused by endothelial damage can lead to decreased renal perfusion and increased serum creatinine levels, which are associated with more advanced disease and poor clinical outcomes (6). A decrease in platelet count may reflect platelet consumption due to endothelial damage and microthrombotic processes and may be associated with worse clinical outcomes. The combined assessment of LDH, serum creatinine, and platelet count allows the EASIX score to reflect both the biological characteristics of the tumor and the patient's systemic status within a single index (7). The EASIX score was initially developed to predict the risk of developing transplant-associated thrombotic microangiopathy (TA-TMA) in patients undergoing allogeneic hematopoietic stem cell transplantation (allo-HSCT), and subsequent studies have shown it to have prognostic value in predicting acute graft-versus-host disease (aGVHD), sinusoidal obstruction syndrome (SOS), and other transplantation-related complications, as well as overall survival (8, 9). In a similar study conducted in patients with primary central nervous system lymphoma (PCNSL), high EASIX was identified as the most important predictor of both overall survival (OS) and progression-free survival (PFS) (10).

Subsequent studies have demonstrated the prognostic value of EASIX not only in hematological malignancies but also in various solid tumors. Similarly, in small cell lung cancer, high EASIX levels have been indicated as an independent prognostic factor for worse progression-free and overall survival, regardless of stage (11). RCC is a highly vascular malignancy characterized by marked angiogenesis, tumor-related inflammation, and microvascular remodeling. Given that endothelial dysfunction plays a significant role in tumor progression, metastatic spread, and treatment response, it is thought that the EASIX score, reflecting endothelial activation and systemic stress, may also provide prognostic information in patients with metastatic renal cell carcinoma (mRCC) (12). However, to our knowledge, there are no studies evaluating the prognostic value of the EASIX score in patients with mRCC. Therefore, this study aimed to investigate the association between baseline EASIX and overall survival in patients with mRCC receiving systemic treatment and evaluate its potential utility as a prognostic biomarker.

## 2 Materials and methods

### 2.1 Study design

This retrospective study was conducted at the Medical Oncology Clinic of the University of Health Sciences, Sultan II. Abdulhamid Han Training and Research Hospital to evaluate the prognostic value of the Endothelial Activation and Stress Index (EASIX) in patients with histopathologically confirmed mRCC. Patients who were diagnosed with mRCC between January 2016 and December 2025 and for whom complete clinical, pathological, laboratory, and follow-up data were available were included in the study. Demographic characteristics (age, sex, performance status), clinicopathological features [TNM stage, histological subtype, Fuhrman/International Society of Urological Pathology (ISUP) nuclear grade], laboratory parameters, treatment modalities, and follow-up information were retrospectively retrieved from the hospital electronic medical records. The study was conducted in accordance with the principles of the Declaration of Helsinki and was approved by the Non-Interventional Scientific Research Ethics Committee of the University of Health Sciences, Hamidiye (Project No: 25/880, Decision No: 22/4, Approval Date: 25 December 2025). Patients were excluded if the laboratory parameters required to calculate the EASIX score (serum lactate dehydrogenase, serum creatinine, and platelet count) before the initiation of first-line systemic treatment were missing or if follow-up data were unavailable. In addition, patients with synchronous malignancies, active sepsis, or other severe active infectious or inflammatory diseases that could significantly affect laboratory parameters were not included in the analysis. The primary endpoint of the study was overall survival (OS), defined as the time from the date of diagnosis to death from any cause or last follow-up. The EASIX score at diagnosis was calculated using serum lactate dehydrogenase (LDH), serum creatinine, and platelet count. The EASIX score was calculated according to the following formula:

$$EASIX\;=\;(LDH\;[U/L]\;\times \;serum\;creatinine\;[mg/dL])\;/\;platelet\;count\;(10^{9}/L).$$

### 2.2 Statistical analysis

Descriptive analyses were performed to summarize the demographic, clinical, laboratory, and inflammatory characteristics of the study population. The distribution of continuous variables was assessed using the Kolmogorov–Smirnov test, Shapiro–Wilk test, histogram inspection, skewness, and kurtosis. Categorical variables were presented as number (n) and percentage (%). Continuous variables with a normal distribution were expressed as mean ± standard deviation (SD), whereas non-normally distributed variables were presented as median (25th–75th percentiles). Comparisons between the low- and high-EASIX groups were performed using the independent-samples Student's *t* test for normally distributed continuous variables and the Mann–Whitney *U* test for non-normally distributed continuous variables. Categorical variables were compared using chi-square tests. Exploratory receiver operating characteristic (ROC) curve analyses based on observed mortality status were performed to evaluate the discriminatory ability of EASIX, neutrophil-to-lymphocyte ratio (NLR), monocyte-to-lymphocyte ratio (MLR), platelet-to-lymphocyte ratio (PLR), and systemic immune-inflammation index (SII) and to identify a cohort-derived EASIX cut-off value. The cut-off value was determined using the maximum Youden index, and the corresponding area under the curve (AUC), sensitivity, specificity, and 95% confidence interval (CI) were calculated. Based on the identified cut-off value, the EASIX score was categorized into two groups (≤ 1.293 and > 1.293). Kaplan–Meier survival analyses were performed to estimate survival according to the categorized EASIX score, and survival curves were compared using the log-rank test. Univariate Cox proportional hazards regression analyses were performed to evaluate the association between each variable and overall survival. Additionally, EASIX was analyzed as a continuous variable in a separate univariate Cox regression model to assess its association with overall survival without relying on the selected cut-off value.

Variables with a *p* value < 0.05 in the univariate analyses were subsequently included in the multivariate Cox proportional hazards regression model. The number of variables included in the model was kept limited, taking into consideration the available sample size and number of observed events. Additional multivariate Cox regression analyses incorporating ECOG performance status were performed in patients with available ECOG data. EASIX was evaluated separately as a categorical and a continuous variable. Furthermore, univariable Cox regression analyses were performed separately for the individual components of EASIX (LDH, serum creatinine, and platelet count) to assess their associations with overall survival. These component analyses were conducted separately from the primary prognostic model, as the individual components are mathematically incorporated into the EASIX score. Hazard ratios (HRs) and their corresponding 95% confidence intervals (CIs) were reported. A two-sided *p* value < 0.05 was considered statistically significant. All statistical analyses were performed using IBM SPSS Statistics for Mac, version 27.0 (IBM Corp., Armonk, NY, USA).

## 3 Results

A total of 143 patients with mRCC were included in the study. The mean age at diagnosis was 59.86 ± 12.64 years, and 111 (77.6%) patients were male. At least one comorbidity was present in 89 (62.2%) patients. ISUP grade was available for 109 patients; grade 3 was observed in 50 (45.9%) patients, while grade 4 was observed in 16 (14.7%) patients. *De novo* metastatic disease was present in 129 (90.2%) patients, whereas 14 (9.8%) had recurrent disease. The lung was the most common metastatic site, occurring in 56 (39.2%) patients. First-line treatment consisted of sunitinib in 126 (88.1%) patients, pazopanib in 15 (10.5%), and cabozantinib in 2 (1.4%). During follow-up, 74 (51.7%) patients died. The median EASIX score was 1.43 [0.87–2.53]. Other laboratory parameters are presented in Table 1.

**Table 1:** Baseline demographic and clinical characteristics of patients with metastatic renal cell carcinoma.

Variable	Total (*n* = 143)
Age at diagnosis, years^a^	59.86 ± 12.64
Sex, n (%)	
Male	111 (77.6)
Female	32 (22.4)
BMI, kg/m^2^^b^	23.72 [21.43–25.60]
ECOG performance status, n (%)	
0	71 (71.0)
≥ 1	29 (29.0)
Comorbidity, n (%)	
Absent	54 (37.8)
Present	89 (62.2)
Histological subtype, n (%)	
Clear cell RCC	116 (81.1)
Non-clear cell RCC	27 (18.9)
ISUP grade, n (%)	
Low (1­–2)	43 (39.4)
High (3–4)	66 (60.6)
Sites of metastasis, n (%)	
Lung	56 (39.2)
Bone	47 (32.9)
Liver	19 (13.3)
Adrenal gland	37 (25.9)
Other	54 (37.8)
Disease presentation, n (%)	
*de novo* metastatic disease	129 (90.2)
Recurrence	14 (9.8)
First-line treatment	
Sunitinib	126 (88.1)
Pazopanib	15 (10.5)
Cabozantinib	2 (1.4)
Laboratory parameters	
LDH (U/L)^b^	274 [208–329]
Creatinine (mg/dL)^b^	1.12 [0.86–1.34]
Platelet (×10^9^/L)^b^	204 [161–256]
Neutrophil (×10^3^/µL)^b^	5.21 [3.62–7.46]
Lymphocyte (×10^3^/µL)^b^	1.77 [1.21–2.43]
Monocyte (×10^3^/µL)^b^	0.49 [0.36–0.69]
SII^b^	528.25 [352.36–1037.26]
NLR^b^	2.61 [1.68–4.93]
MLR^b^	0.29 [0.17–0.45]
PLR^b^	111.79 [87.03–169.34]
EASIX^b^	1.43 [0.87–2.53]
Survival status, n (%)	
Alive	69 (48.3)
Dead	74 (51.7)

BMI, body mass index; ECOG, Eastern Cooperative Oncology Group; ISUP, International Society of Urological Pathology; RCC, renal cell carcinoma; LDH, lactate dehydrogenase; EASIX, Endothelial Activation and Stress Index; SII, systemic immune-inflammation index; NLR, neutrophil-to-lymphocyte ratio; MLR, monocyte-to-lymphocyte ratio; PLR, platelet-to-lymphocyte ratio; IQR, interquartile range. Data are presented as mean ± standard deviation (SD), median [interquartile range (IQR)], or n (%), as appropriate.

a Mean ± SD.

b Median (IQR).

ECOG performance status was available for 100 patients, and ISUP grade was available for 109 patients. Percentages were calculated using available data.

Exploratory receiver operating characteristic (ROC) curve analyses based on observed mortality status were performed to evaluate the discriminatory ability of EASIX, NLR, MLR, PLR, and SII. The area under the curve (AUC) for EASIX was 0.855 (95% CI: 0.796–0.915, *p* < 0.001). The cohort-derived EASIX cut-off value, determined using Youden's index, was 1.293, yielding a sensitivity of 82.40% and a specificity of 69.60% (Figure 1). In contrast, the AUC for NLR was 0.534 (95% CI: 0.438–0.630, *p* = 0.482). The cut-off value determined using Youden's index was 2.606, yielding a sensitivity of 52.70% and a specificity of 52.20%. Similarly, the AUC for MLR was 0.523 (95% CI: 0.427–0.619, *p* = 0.638). The cut-off value was 0.291, with a sensitivity of 52.70% and a specificity of 53.60%. The AUC for SII was 0.434 (95% CI: 0.339–0.529, *p* = 0.173), with an cut-off value of 540.241, a sensitivity of 44.60%, and a specificity of 44.90%. The AUC for PLR was 0.454 (95% CI: 0.360–0.549, *p* = 0.347), with an cut-off value of 111.523, a sensitivity of 45.90%, and a specificity of 44.90%.

**Figure 1:**
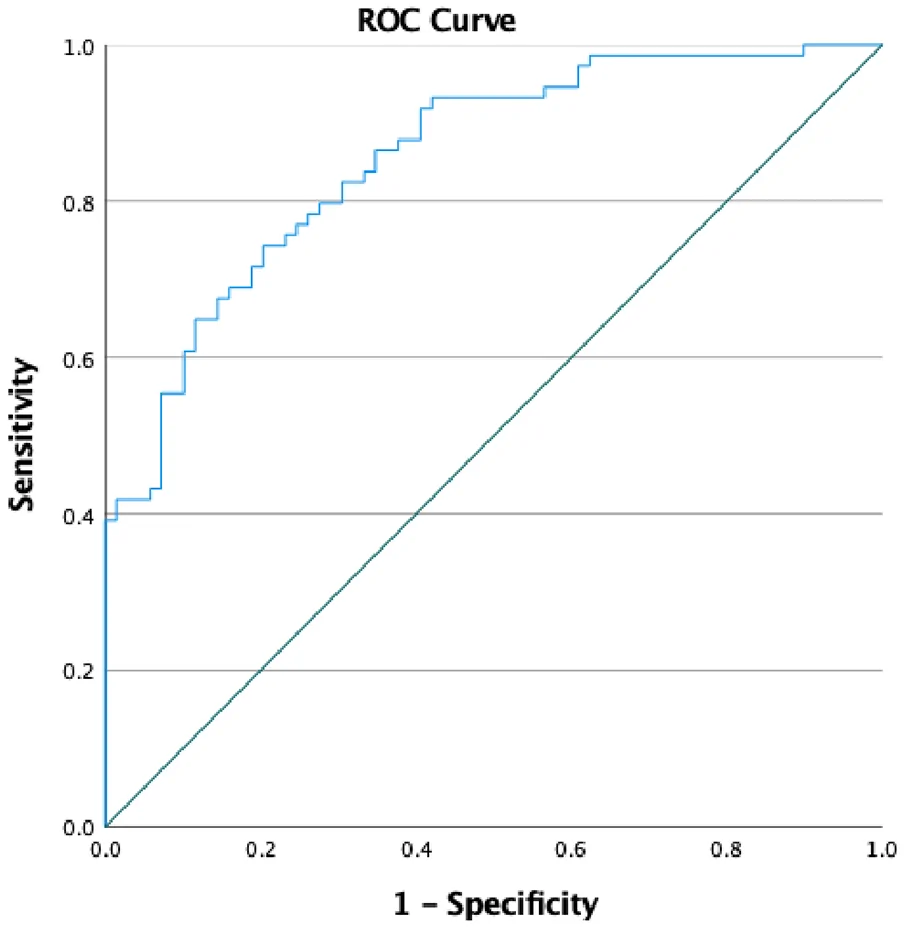
Receiver operating characteristic (ROC) curve of EASIX for predicting mortality.

When the EASIX groups defined according to the cut-off value determined by ROC analysis were compared, age was significantly higher in the high EASIX group (*p* = 0.004). In addition, serum creatinine and LDH levels were significantly higher, whereas platelet count was significantly lower in the high EASIX group (all *p* < 0.001). No significant differences were observed between the groups in terms of sex, ECOG performance status, *de novo* metastatic disease, metastatic sites, ISUP grade, histological subtype, or inflammatory indices (SII, PLR, NLR, and MLR) (all *p* > 0.05). Although bone metastasis was more frequent in the high EASIX group, the difference did not reach statistical significance (39.0% vs. 24.6%; *p* = 0.069). In contrast, the mortality rate was significantly higher in the high EASIX group (74.4% vs. 21.3%; *p* < 0.001) (Table 2).

**Table 2:** Clinical characteristics and inflammatory indices according to EASIX groups.

Variable	Low EASIX (≤ 1.293) (*n* = 61)	High EASIX (> 1.293) (*n* = 82)	*p* value
Age (years)^a^	56.15 ± 12.98	62.62 ± 11.71	**0** **.** **004**
Sex, n (%)			0.340
Male	45 (73.8)	66 (80.5)	
Female	16 (26.2)	16 (19.5)	
ECOG performance status, n (%)			0.554
0	34 (73.9)	37 (68.5)	
≥ 1	12 (26.1)	17 (31.5)	
Creatinine (mg/dL)^b^	0.92 [0.64–1.13]	1.21 [1.05–1.60]	**<0.001**
LDH (U/L)^b^	213 [182.50–276.50]	316 [246.50–370.75]	**<0.001**
Platelet (×10⁹/L)^b^	246 [207.00–301.00]	185 [143.25–214.25]	**<0.001**
SII^b^	649.41 [360.66–1337.36]	483.21 [323.55–936.44]	0.099
PLR^b^	117.18 [92.41–184.32]	108.39 [77.11–154.97]	0.093
NLR^b^	2.42 [1.54–4.73]	2.96 [1.95–5.16]	0.319
MLR^b^	0.27 [0.17–0.42]	0.32 [0.17–0.47]	0.421
*De novo* metastatic disease, n (%)			0.987
Yes	55 (90.2)	74 (90.2)	
No	6 (9.8)	8 (9.8)	
Liver metastasis, n (%)			0.294
No	55 (90.2)	69 (84.1)	
Yes	6 (9.8)	13 (15.9)	
Lung metastasis, n (%)			0.178
No	41 (67.2)	46 (56.1)	
Yes	20 (32.8)	36 (43.9)	
Bone metastasis, n (%)			0.069
No	46 (75.4)	50 (61.0)	
Yes	15 (24.6)	32 (39.0)	
ISUP grade, n (%)			0.462
Low (Grade 1–2)	20 (43.5)	23 (36.5)	
High (Grade 3–4)	26 (56.5)	40 (63.5)	
Histological subtype, n (%)			0.512
Clear cell	51 (83.6)	65 (79.3)	
Non-clear cell	10 (16.4)	17 (20.7)	
Vital status, n (%)			**<0.001**
Alive	48 (78.7)	21 (25.6)	
Dead	13 (21.3)	61 (74.4)	

Continuous variables are presented as mean ± standard deviation (SD) or median [interquartile range (IQR)], as appropriate. Categorical variables are presented as number (percentage).

a Mean ± SD.

b Median (IQR).

EASIX, Endothelial Activation and Stress Index; ECOG, Eastern Cooperative Oncology Group; LDH, lactate dehydrogenase; SII, systemic immune-inflammation index; PLR, platelet-to-lymphocyte ratio; NLR, neutrophil-to-lymphocyte ratio; MLR, monocyte-to-lymphocyte ratio; ISUP, International Society of Urological Pathology. ECOG performance status was available for 100 patients, and ISUP grade was available for 109 patients. Percentages were calculated using available data.

Bold values indicate statistically significant results (*p* < 0.05).

Overall survival (OS) according to EASIX score groups was evaluated using the Kaplan–Meier method. The median OS for the entire cohort was 37.9 months (95% CI: 29.0–46.9). In the high EASIX group (> 1.293), the median OS was 22.0 months (95% CI: 14.8–29.2), whereas the median OS was not reached in the low EASIX group (≤ 1.293). A statistically significant difference in OS was observed between the groups (log-rank *χ*^2^ = 30.986; *p* < 0.001). During follow-up, a total of 74 deaths occurred. There were 13 deaths and 48 censored cases in the low EASIX group, compared with 61 deaths and 21 censored cases in the high EASIX group. Overall survival curves according to EASIX score groups and the numbers of patients at risk are shown in Figure 2.

**Figure 2:**
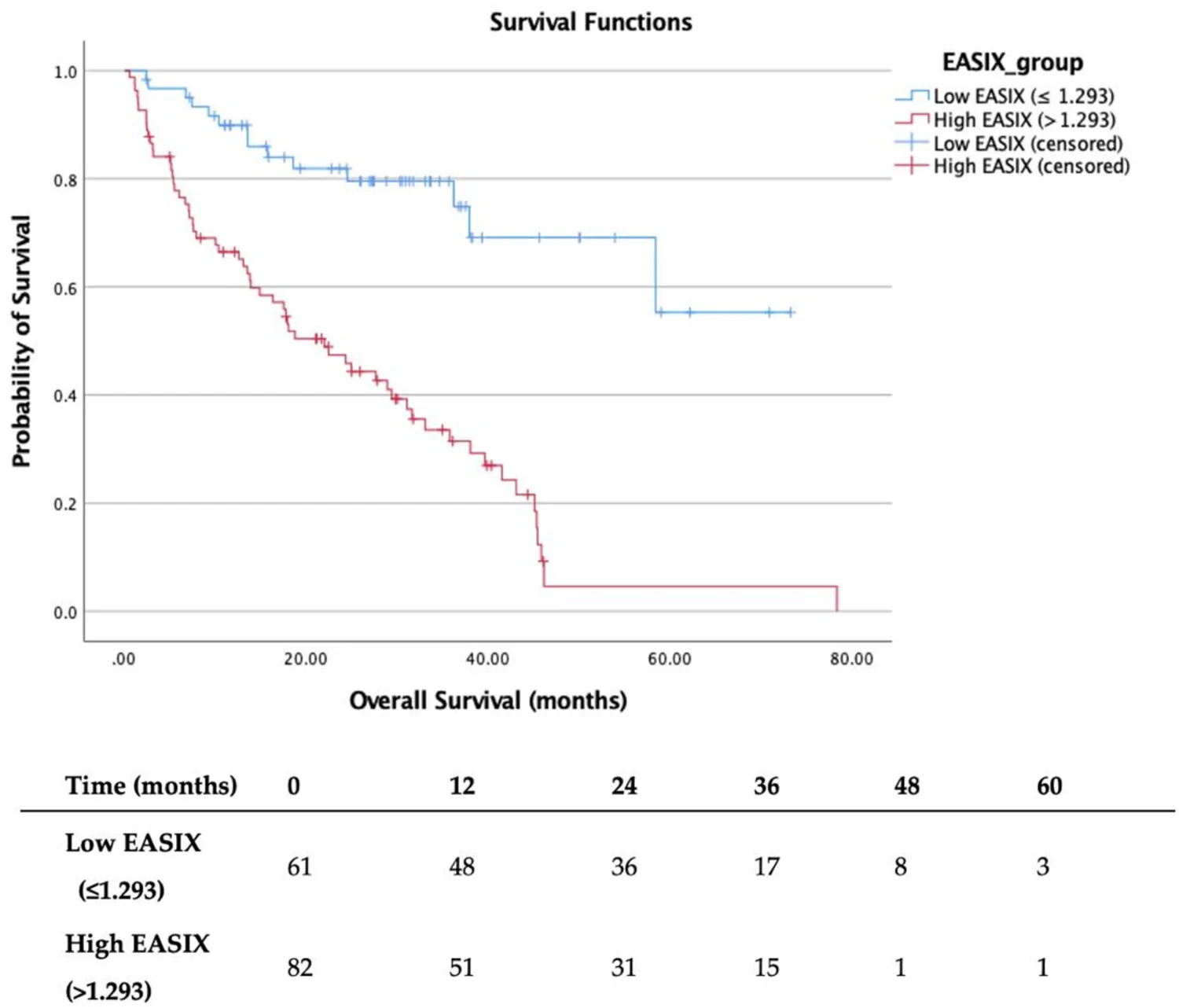
Kaplan–meier curves for overall survival according to EASIX score groups. The numbers at risk are presented below the Kaplan–Meier curves.

When factors associated with overall survival were evaluated, univariate Cox regression analysis showed that a high EASIX score was associated with an approximately 4.8-fold higher risk of death compared with a low EASIX score (HR: 4.794; 95% CI: 2.613–8.793; *p* < 0.001). Liver metastasis (HR: 2.843; 95% CI: 1.606–5.032; *p* < 0.001) and lung metastasis (HR: 1.945; 95% CI: 1.225–3.088; *p* = 0.005) were also significantly associated with overall survival. In contrast, sex, histological subtype, ISUP grade, and metastatic presentation were not significantly associated with overall survival all *p* > 0.05). In multiviate Cox regression analysis, a high EASIX score remained significantly associated with overall survival after adjustment for age, lung metastasis, and liver metastasis (HR: 4.031; 95% Cl: 2.146–7.572; *p* < 0.001).

When EASIX was analyzed as a continuous variable in a separate univariate Cox regression model, higher EASIX values were significantly associated with an increased hazard of death (HR: 1.345 per one-unit increase; 95% CI: 1.242–1.457; *p* < 0.001). Liver metastasis (HR: 2.054; 95% CI: 1.152–3.663; *p* = 0.015) and lung metastasis (HR: 1.618; 95% CI: 1.015–2.580; *p* = 0.043) were also identified as independent prognostic factors for overall survival. In contrast, age was not statistically significant in the multivariate analysis (HR: 1.012; 95% CI: 0.989–1.035; *p* = 0.302) (Table 3). In addition, exploratory univariate Cox regression analyses of the individual EASIX components showed that LDH (HR: 1.004; 95% Cl: 1.003–1.005; *p* < 0.001), serum creatinine (HR: 1.431; 95% Cl:1.227- 1.669; *p* < 0.001), and platelet count (HR: 0.994; 95% Cl: 0.990–0.998; *p* = 0.002) were each significantly associated with overall survival (Supplementary Table S1). In sensitivity analyses including 100 patients with available ECOG performance status data and 49 deaths, high EASIX remained significantly associated with worse overall survival after adjustment for age, ECOG performance status, lung metastasis, and liver metastasis (HR: 4.149; 95% CI: 1.989–8.653; *p* < 0.001). Similarly, when EASIX was evaluated as a continuous variable, higher EASIX values were significantly associated with an increased hazard of death (HR: 1.431 per one-unit increase; 95% CI: 1.278–1.602; *p* < 0.001) (Supplementary Table S2).

**Table 3:** Univariate and multivariate cox regression analysis for overall survival.

Variable	Univariate HR (95% CI)	*p* value	Multivariate HR (95% CI)	*p* value
Age	1.030 (1.009–1.052)	**0.004**	1.012 (0.989–1.035)	0.302
Sex (Male vs. Female)	0.724 (0.403–1.302)	0.281		
Histology (Non-clear vs. Clear)	1.11 (0.59–2.07)	0.749		
ISUP grade (High vs. Low)	1.544 (0.850–2.804)	0.154	-	-
Metastatic presentation (Recurrent vs. *De novo*)	0.561 (0.253–1.246)	0.156		
Lung metastasis	1.945 (1.225–3.088)	**0.005**	1.618 (1.015–2.580)	**0.043**
Liver metastasis	2.843 (1.606–5.032)	**<0.001**	2.054 (1.152–3.663)	**0.015**
High EASIX vs. Low EASIX	4.794 (2.613–8.793)	**<0.001**	4.031 (2.146–7.572)	**<0.001**

HR, hazard ratio; CI, confidence interval; EASIX, Endothelial Activation and Stress Index; ECOG, Eastern Cooperative Oncology Group performance status; ISUP, International Society of Urological Pathology.

Bold values indicate statistically significant results (*p* < 0.05).

## 4 Discussion

In this study, 143 patients with mRCC were evaluated according to their EASIX scores at diagnosis. Our findings showed that a high EASIX score was significantly associated with worse overall survival, and this association persisted in multivariate Cox regression analyses, including sensitivity analyses incorporating ECOG performance status. In addition, exploratory ROC analysis showed that EASIX had statistically significant discriminatory ability for observed mortality in this cohort, whereas conventional inflammatory indices, including NLR, MLR, PLR, and SII, did not reach statistical significance. These findings suggest that EASIX may be an easily calculated biomarker with potential utility in risk stratification and prognostic evaluation in patients with mRCC, although further validation is needed before its routine clinical application.

In recent years, several studies have supported the use of EASIX as a prognostic biomarker not only in hematological malignancies but also in solid tumors. Although the prognostic value of EASIX has not yet been evaluated specifically in patients with mRCC, similar findings across different tumor types suggest that its prognostic relevance may extend to different malignancies. One of the few studies supporting the prognostic significance of EASIX in urological malignancies is the multicenter retrospective study by Gu et al. in patients who underwent radical nephroureterectomy for upper urinary tract urothelial carcinoma. In that study, a higher prediagnostic EASIX score was associated with shorter recurrence-free survival and overall survival, and EASIX remained independently associated with both outcomes after adjustment for clinicopathological variables. Although this patient population differs from our mRCC cohort in terms of tumor histology, disease stage, and treatment approach, the results support the view that EASIX may provide prognostic information as a composite indicator of tumor biology, organ function, and systemic stress in urothelial and renal malignancies (13). Similarly, Park et al. reported that a high EASIX score was associated with shorter progression-free survival and overall survival in patients with newly diagnosed diffuse large B-cell lymphoma and retained its independent prognostic significance for both progression-free survival and overall survival in multivariate analysis (14). A similar survival association was observed in our study; median overall survival was 22.0 months in the high EASIX group, whereas median overall survival was not reached in the low EASIX group (*p* < 0.001). A high EASIX score was associated with an approximately 4.8-fold increased risk of death in univariate Cox regression analysis (HR: 4.794; 95% CI: 2.613–8.793; *p* < 0.001) and remained significantly associated with overall survival after adjustment for the clinical variables included in the multivariate model (HR: 4.031; 95% CI: 2.146–7.572; *p* < 0.001). The association between higher EASIX values and poorer overall survival was also observed when EASIX was analyzed as a continuous variable, suggesting that this relationship was not solely dependent on the selected cut-off value.

One of the notable findings of our study was that EASIX demonstrated statistically significant discriminatory ability for observed mortality, whereas the other inflammatory indices evaluated in the same cohort did not reach statistical significance. In the exploratory ROC analysis, EASIX showed good discrimination of observed mortality status (AUC: 0.855; 95% CI: 0.796–0.915; *p* < 0.001), suggesting its potential utility as a prognostic biomarker in patients with mRCC. However, this finding does not imply that these inflammatory indices lack the prognostic value reported in previous studies. Indeed, in a meta-analysis of patients with mRCC conducted by Xu et al., a high SII was found to be an adverse prognostic factor for both overall survival and progression-free survival (15). Similarly, a meta-analysis of patients with RCC conducted by Wang et al. showed that high PLR values were associated with poorer survival outcomes in patients with RCC (16).

In our study, the significant association between EASIX and overall survival, together with the absence of significant differences in NLR, MLR, PLR, and SII between the low and high EASIX groups, suggests that the prognostic value of EASIX may not solely reflect systemic inflammation. This difference may be related to the ability of EASIX to simultaneously reflect different biological processes, such as tumor burden, cellular damage, organ function, and endothelial and thromboinflammatory activity, through its components of LDH, creatinine, and platelet count. In this respect, the prognostic association observed for EASIX in our study may be related to its ability to reflect different biological processes involving both the tumor and the host, in addition to inflammation (17). Although EASIX has been proposed as a marker of endothelial activation and stress, its individual components may reflect different clinical and biological processes. LDH may reflect tumor burden and cellular injury, serum creatinine may reflect renal function and comorbidities, and platelet count may be influenced by systemic inflammation and other hematological conditions. In our study, LDH, serum creatinine, and platelet count were each significantly associated with overall survival in separate univariate Cox regression analyses. These findings suggest that the prognostic association of EASIX may reflect multiple biological processes rather than endothelial activation or dysfunction alone. Regarding the conventional inflammatory indices, several factors may explain why our findings differed from those reported in previous studies. The fact that these indices are calculated only from peripheral blood cells and that these parameters can be affected by various clinical conditions such as infection and inflammation may contribute to the difference between the results. In addition, the heterogeneity of cut-off values and patient populations and clinical characteristics used in previous studies may cause the prognostic performance of these markers to vary between studies. Indeed, meta-analyses evaluating NLR and PLR have reported that heterogeneity between studies is a factor that should be taken into account in interpreting the results (18, 19).

When clinicopathological characteristics were compared between the EASIX groups, no significant differences were observed in histological subtype or ISUP grade. However, given the established prognostic importance of both variables in RCC, these findings should be interpreted in the context of the existing literature. In a cohort of 10,105 patients with mRCC, Dudani et al. reported that patterns of metastatic spread differed significantly among clear cell, papillary, and chromophobe RCC and that survival outcomes also varied according to histological subtype. These findings highlight the substantial biological and clinical heterogeneity among histological subtypes of mRCC (20). In the multicenter study by Choi et al., which included 911 patients with mRCC, median overall survival was significantly shorter in patients with high WHO/ISUP grade than in those with low grade. Although high grade was identified as an independent risk factor for overall survival in multivariate analysis, its prognostic effect varied according to T and N stage, suggesting that the prognostic value of tumor grade may differ depending on the clinical context (21). In contrast to these findings in the literature, no significant differences were observed between the high and low EASIX groups in terms of histological subtype or ISUP grade in our study. In addition, neither variable was significantly associated with overall survival in univariate Cox regression analysis. This difference may be related to the characteristics of our cohort. The predominance of clear cell histology (81.1%), with non-clear cell cases accounting for only 18.9% of the cohort, may have made it difficult to detect prognostic differences between histological subtypes. Similarly, ISUP grade data were not available for the entire cohort, which may have limited the ability to detect prognostic differences between grades. In addition, the fact that the WHO/ISUP grading system may have different prognostic implications across histological subtypes should be taken into account when interpreting this finding.

At the same time, when other findings related to overall survival other than EASIX were evaluated in our study, it was shown that liver and lung metastasis had prognostic importance in both univariate and multivariate analyses. In particular, the fact that liver metastasis was associated with an approximately two-fold increased risk of death (HR: 2.054; 95% CI: 1.152–3.663; *p* = 0.015) supports the prognostic importance of the metastatic site in RCC, as in the literature. In Lee et al.'s multicenter study including 1,761 mRCC patients, cancer-specific survival varied significantly depending on the site of metastasis; It has been reported that the median cancer-specific survival in patients with liver metastases is one of the shortest at 13.9 months, whereas it reaches 29.1 months in patients with lung metastases. In that study, liver metastasis was implicated as one of the metastatic sites associated with the most unfavorable cancer-specific survival outcomes (22). However, unlike the findings reported by Lee et al., lung metastasis was also associated with worse survival in our study. This difference may be related to differences in patient population characteristics, metastatic disease burden, systemic treatments, and the endpoints evaluated.

In our study, age was associated with overall survival in univariate analysis but lost its independent prognostic significance in multivariate analysis when evaluated together with the EASIX score and metastatic sites. Findings regarding the effect of age on survival in mRCC are heterogeneous in the literature. For example, in the analysis by Hermansen et al. of patients with mRCC, age was not independently associated with overall survival in multivariate analysis when evaluated either as a continuous variable or using different age thresholds (23). In contrast, the large population-based analysis by Yue et al. reported that age was an independent prognostic factor in patients with mRCC. These differing results suggest that the prognostic effect of chronological age should be considered together with patient clinical characteristics, disease burden, and treatment approach rather than evaluated in isolation (24).

One of the important aspects of our study was the evaluation of EASIX in relation to overall survival, together with an exploratory assessment of its discriminatory ability for observed mortality alongside conventional inflammatory indices such as NLR, MLR, PLR, and SII. EASIX is calculated using LDH, creatinine, and platelet count, which are readily available in routine clinical practice, and does not require additional laboratory testing or costs, providing an important advantage in terms of its ease of applicability. The association between EASIX and overall survival in both univariate and multivariate analyses supports its potential role as a prognostic biomarker in patients with mRCC, although its clinical utility requires further evaluation. This association was also observed in sensitivity analyses incorporating ECOG performance status, with EASIX remaining significantly associated with overall survival when evaluated as either a categorical or a continuous variable.

When interpreting our findings, it should be considered that this was a retrospective, single-center study with a limited number of patients, which may have limited the ability to detect the prognostic effects of clinical and pathological variables in small subgroups. The high proportion of patients with *de novo* metastatic disease in our cohort may also limit the generalizability of our findings to the broader mRCC population, particularly to patients who develop metastatic recurrence after initially localized disease. Most patients in our cohort received sunitinib as first-line treatment, reflecting the reimbursement policies and access to systemic therapies in our country during the study period. Although immune checkpoint inhibitor-based combinations are currently the preferred first-line treatment for many patients with mRCC, access to these treatments remains limited in some healthcare systems, where tyrosine kinase inhibitors continue to be used as first-line therapy. Therefore, our findings may remain clinically relevant in settings with restricted access to immunotherapy-based treatments. Nevertheless, the predominance of sunitinib-treated patients in our cohort limits the generalizability of our results to patients receiving contemporary immunotherapy-based combinations. Further studies are needed to validate the prognostic value of EASIX in patients receiving current systemic treatment regimens. In addition, due to the limited number of patients in our study, the non-clear cell RCC subgroup, which included papillary, chromophobe, and other rare subtypes, represented a heterogeneous group. The evaluation of these subtypes under a single “non-clear cell” category may have limited the assessment of potential prognostic differences among individual subtypes. Similarly, due to the retrospective nature of the study, ECOG performance status and ISUP grade data were not available for all patients. The incomplete availability of established prognostic factors, including ECOG performance status and ISUP grade, may have limited their assessment in relation to overall survival. Although sensitivity analyses incorporating ECOG performance status were performed, the reduced sample size and potential selection bias due to missing data should be considered when interpreting these results.

In addition, incomplete data prevented the reliable assessment of IMDC risk classification in all patients. Therefore, IMDC risk groups could not be included in the multivariate Cox regression analysis. Although EASIX remained significantly associated with overall survival after adjustment for the clinical variables included in our model, the inability to adjust for IMDC risk classification limits the interpretation of its independent prognostic value. Further studies incorporating established prognostic models are needed to determine whether EASIX provides additional prognostic information beyond conventional risk stratification. Another limitation of our study is that the EASIX cut-off value of 1.293 was derived using ROC analysis and the Youden index and subsequently evaluated in the same patient cohort. This approach may have led to an overestimation of its prognostic performance. Furthermore, the absence of internal and external validation limits the generalizability of the identified cut-off value. Therefore, both the AUC of 0.855 and the derived cut-off value should be considered exploratory findings that require further validation in independent patient cohorts. Moreover, conventional ROC analysis does not account for differences in follow-up duration or censoring. Therefore, the resulting AUC values reflect discrimination based on observed mortality status rather than survival prediction at specific time points. This methodological limitation should be taken into consideration when interpreting the findings of our study.

In conclusion, our study suggests that EASIX may be an easily applicable prognostic marker associated with overall survival in patients with mRCC, predominantly treated with first-line sunitinib. The significant discriminatory ability of EASIX for observed mortality in exploratory ROC analysis, together with its calculation from routine laboratory parameters, supports further investigation of its potential clinical utility. The EASIX cut-off value of 1.293 was derived from the present cohort, and its validity in independent patient populations remains to be established. The prognostic value of EASIX should be prospectively validated in larger, independent mRCC cohorts, particularly in patients receiving contemporary immunotherapy-based treatment combinations.

## Data Availability

The raw data supporting the conclusions of this article will be made available by the authors, without undue reservation.

## References

[1] Bray F Laversanne M Sung H Ferlay J Siegel RL Soerjomataram I. Global cancer statistics 2022: GLOBOCAN estimates of incidence and mortality worldwide for 36 cancers in 185 countries. CA Cancer J Clin. (2024) 74(3):229–63. 10.3322/caac.21834 38572751

[2] Bukavina L Bensalah K Bray F Carlo M Challacombe B Karam JA. Epidemiology of renal cell carcinoma: 2022 update. Eur Urol. (2022) 82(5):529–42. 10.1016/j.eururo.2022.08.019 36100483

[3] Capitanio U Bensalah K Bex A Boorjian SA Bray F Coleman J. Epidemiology of renal cell carcinoma. Eur Urol. (2019) 75(1):74–84. 10.1016/j.eururo.2018.08.036 30243799 PMC8397918

[4] Bex A Ghanem YA Albiges L Bonn S Campi R Capitanio U. European association of urology guidelines on renal cell carcinoma: the 2025 update. Eur Urol. (2025) 87(6):683–96. 10.1016/j.eururo.2025.02.020 40118739

[5] Forkasiewicz A Dorociak M Stach K Szelachowski P Tabola R Augoff K. The usefulness of lactate dehydrogenase measurements in current oncological practice. Cell Mol Biol Lett. (2020) 25:35. 10.1186/s11658-020-00228-7 32528540 PMC7285607

[6] Yang M Zhang Q Ruan G-T Tang M Zhang X Song M-M. Association between serum creatinine concentrations and overall survival in patients with colorectal cancer: a multicenter cohort study. Front Oncol. (2021) 11:710423. 10.3389/fonc.2021.710423 34692487 PMC8529284

[7] Maneta E Aivalioti E Tual-Chalot S Emini Veseli B Gatsiou A Stamatelopoulos K. Endothelial dysfunction and immunothrombosis in sepsis. Front Immunol. (2023) 14:1144229. 10.3389/fimmu.2023.1144229 37081895 PMC10110956

[8] Jodele S Zhang K Zou F Laskin B Dandoy CE Myers KC. The genetic fingerprint of susceptibility for transplant-associated thrombotic microangiopathy. Blood. (2016) 127(8):989–96. 10.1182/blood-2015-08-663435 26603840 PMC4828073

[9] Pedraza A Salas MQ Rodríguez-Lobato LG Escribano-Serrat S Suárez-Lledo M Martínez-Cebrian N. Easix score correlates with endothelial dysfunction biomarkers and predicts risk of acute graft-versus-host disease after allogeneic transplantation. Transplant Cell Ther. (2024) 30(2):187.e1–187.e12. 10.1016/j.jtct.2023.11.016 38000709

[10] Zhao H Kong L Wang J Gao W Zhu W Zhao J. The endothelial activation and stress index (EASIX) at diagnosis is associated with survival in primary central nervous system lymphoma. Br J Haematol. (2026) 209:1047–56. 10.1111/bjh.70641 42389864 PMC13570180

[11] Go S-I Park S Kang MH Kim H-G Kang JH Kim JH. Endothelial activation and stress index (EASIX) as a predictive biomarker in small cell lung cancer. Cancer Biomark. (2022) 35(2):217–25. 10.3233/CBM-220032 36120771 PMC12364233

[12] Guillaume Z Auvray M Vano Y Oudard S Helley D Mauge L. Renal carcinoma and angiogenesis: therapeutic target and biomarkers of response in current therapies. Cancers (Basel). (2022) 14(24):6167. 10.3390/cancers14246167 36551652 PMC9776425

[13] Gu JS Ryu JW Yu SH Chung HS Hwang JE Bae WK. Prognostic value of the endothelial activation and stress index in patients with upper tract urothelial cancer undergoing radical nephroureterectomy. Investig Clin Urol. (2022) 63(6):623–30. 10.4111/icu.20220204 36347551 PMC9643726

[14] Park S Go SI Lee GW. The endothelial activation and stress index (EASIX) score is an independent prognostic factor in patients with diffuse large B-cell lymphoma. BMC Cancer. (2022) 22(1):816. 10.1186/s12885-022-09915-4 35879680 PMC9312320

[15] Xu J Chen P Cao S Hu X Li X. Prognostic value of systemic immune-inflammation index in patients with metastatic renal cell carcinoma treated with systemic therapy: a meta-analysis. Front Oncol. (2024) 14:1404753. 10.3389/fonc.2024.1404753 38962274 PMC11220114

[16] Wang X Su S Guo Y. The clinical use of the platelet to lymphocyte ratio and lymphocyte to monocyte ratio as prognostic factors in renal cell carcinoma: a systematic review and meta-analysis. Oncotarget. (2017) 8(48):84506–14. 10.18632/oncotarget.21108 29137443 PMC5663615

[17] Luft T Benner A Jodele S Dandoy CE Storb R Gooley T. EASIX in patients with acute graft-versus-host disease: a retrospective cohort analysis. Lancet Haematol. (2017) 4(9):e414–23. 10.1016/S2352-3026(17)30108-4 28733186

[18] Zhou X Luo G. A meta-analysis of the platelet-lymphocyte ratio: a notable prognostic factor in renal cell carcinoma. Int J Biol Markers. (2022) 37(2):123–33. 10.1177/03936155221081536 35238678

[19] Hu K Lou L Ye J Zhang S. Prognostic role of the neutrophil-lymphocyte ratio in renal cell carcinoma: a meta-analysis. BMJ Open. (2015) 5(4):e006404. 10.1136/bmjopen-2014-006404 25854964 PMC4390726

[20] Dudani S De Velasco G Wells JC Gan CL Donskov F Porta C. Evaluation of clear cell, papillary, and chromophobe renal cell carcinoma metastasis sites and association with survival. JAMA Netw Open. (2021) 4(1):e2021869. 10.1001/jamanetworkopen.2020.21869 33475752 PMC7821027

[21] Choi J Bang S Suh J Choi CI Song W Yuk HD. Survival pattern of metastatic renal cell carcinoma patients according to WHO/ISUP grade: a long-term multi-institutional study. Sci Rep. (2024) 14:4740. 10.1038/s41598-024-54052-6 38413653 PMC10899595

[22] Lee CH Kang M Kwak C Ko YH Kim JK Park JY. Sites of metastasis and survival in metastatic renal cell carcinoma: results from the Korean renal cancer study group database. J Korean Med Sci. (2024) 39(45):e293. 10.3346/jkms.2024.39.e293 39592128 PMC11596476

[23] Hermansen CK Donskov F. Outcomes based on age in patients with metastatic renal cell carcinoma treated with first line targeted therapy or checkpoint immunotherapy: older patients more prone to toxicity. J Geriatr Oncol. (2021) 12(5):827–33. 10.1016/j.jgo.2020.12.008 33388280

[24] Yue G Deyu L Lianyuan T Fengmin S Mei G Yajun H. Clinical features and prognostic factors of patients with metastatic renal cell carcinoma stratified by age. Aging (Albany NY). (2021) 13(6):8290–305. 10.18632/aging.202637 33686965 PMC8034891

