# Supplementary material for: Endothelial activation and stress index as a prognostic marker in metastatic renal cell carcinoma
Source: Front Med (Lausanne). 2026 Oct 29;13:1966150. doi: 10.3389/fmed.2026.1966150 (PMC13754740; doi:10.3389/fmed.2026.1966150)
Supplement: Supplementary file 1 [file Table1.docx]

Supplementary Material

**Supplementary Table S1.** Univariate Cox regression analysis of individual EASIX components for overall survival.

| **Variable** | **HR (95% CI)** | **p value** |
| --- | --- | --- |
| LDH (U/L) | 1.004 (1.003–1.005) | <0.001 |
| Serum creatinine (mg/dL) | 1.431 (1.227–1.669) | <0.001 |
| Platelet count (×10⁹/L) | 0.994 (0.990–0.998) | 0.002 |

**Abbreviations:** HR, hazard ratio; CI, confidence interval; LDH, lactate dehydrogenase; EASIX, Endothelial Activation and Stress Index
