# Supplementary material for: Endothelial activation and stress index as a prognostic marker in metastatic renal cell carcinoma
Source: Front Med (Lausanne). 2026 Oct 29;13:1966150. doi: 10.3389/fmed.2026.1966150 (PMC13754740; doi:10.3389/fmed.2026.1966150)
Supplement: Supplementary file 2 [file Table2.docx]

Supplementary Material

**Supplementary Table 2.** Sensitivity analyses of the association between EASIX and overall survival using multivariable Cox regression models adjusted for ECOG performance status.

| **Variable** | **Model 1: Categorical EASIX HR (95% CI)** | **p value** | **Model 2: Continuous EASIX HR (95% CI)** | **p value** |
| --- | --- | --- | --- | --- |
| Age | 0.992 (0.963–1.021) | 0.575 | 0.998 (0.967–1.029) | 0.885 |
| ECOG PS (1–2 vs. 0) | 1.894 (0.971–3.694) | 0.061 | 2.029 (0.977–4.213) | 0.058 |
| Lung metastasis (Yes vs. No) | 1.884 (1.049–3.382) | 0.034 | 2.178 (1.167–4.063) | 0.014 |
| Liver metastasis (Yes vs. No) | 2.966 (1.475–5.965) | 0.002 | 3.467 (1.695–7.091) | <0.001 |
| High vs. low EASIX | **4.149 (1.989–8.653)** | **<0.001** | **—** | **—** |
| EASIX (continuous, per unit increase) | **—** | **—** | **1.431 (1.278–1.602)** | **<0.001** |

Note: Both models included 100 patients with available ECOG performance status data, including 49 deaths. Model 1 included EASIX as a dichotomous variable using the cohort-derived cut-off of 1.293. Model 2 included EASIX as a continuous variable. Both models were adjusted for age, ECOG performance status, lung metastasis, and liver metastasis. HR, hazard ratio; CI, confidence interval; ECOG PS, Eastern Cooperative Oncology Group performance status; EASIX, Endothelial Activation and Stress Index.
